# FUNGIpath: a tool to assess fungal metabolic pathways predicted by orthology

**DOI:** 10.1186/1471-2164-11-81

**Published:** 2010-02-01

**Authors:** Sandrine Grossetête, Bernard Labedan, Olivier Lespinet

**Affiliations:** 1 Institut de Génétique et de Microbiologie, Université Paris-Sud 11, CNRS UMR 8621, Bâtiment 400, 91405 Orsay Cedex, France

## Abstract

**Background:**

More and more completely sequenced fungal genomes are becoming available and many more sequencing projects are in progress. This deluge of data should improve our knowledge of the various primary and secondary metabolisms of Fungi, including their synthesis of useful compounds such as antibiotics or toxic molecules such as mycotoxins. Functional annotation of many fungal genomes is imperfect, especially of genes encoding enzymes, so we need dedicated tools to analyze their metabolic pathways in depth.

**Description:**

FUNGIpath is a new tool built using a two-stage approach. Groups of orthologous proteins predicted using complementary methods of detection were collected in a relational database. Each group was further mapped on to steps in the metabolic pathways published in the public databases KEGG and MetaCyc. As a result, FUNGIpath allows the primary and secondary metabolisms of the different fungal species represented in the database to be compared easily, making it possible to assess the level of specificity of various pathways at different taxonomic distances. It is freely accessible at http://www.fungipath.u-psud.fr.

**Conclusions:**

As more and more fungal genomes are expected to be sequenced during the coming years, FUNGIpath should help progressively to reconstruct the ancestral primary and secondary metabolisms of the main branches of the fungal tree of life and to elucidate the evolution of these ancestral fungal metabolisms to various specific derived metabolisms.

## Background

Currently, the Fungi have more published nuclear genome sequences than any other eukaryotic taxonomic group [1]. This relative abundance (28 genomes in May 2009) can be explained by their economic significance and their moderate genome size [Additional File 1]. Since several species are model organisms for fundamental, medical, or agronomical and industrial studies (e.g. *Saccharomyces cerevisiae*, *Candida albicans*, *Yarrowia lipolytica*), fungal genomes seem suitable for large-scale comparative studies, which will allow their evolution to be elucidated [2-4]. Several teams [5-7] have already performed extensive comparisons of a few fungal genomes to predict groups of orthologous proteins, using published methods such as Inparanoid [8], OrthoMCL [9] or TribeMcl [10].

However, current information about the number of fungal enzymes involved in metabolic pathways is rather scanty and is heterogeneously distributed in major public curated databases, both universal (Swiss-Prot [11]) and specialized (KEGG [12] and MetaCyc [13]). To perform an extensive comparison of these various databases containing enzymatic information we propose to identify each enzyme by its ID-EC, which associates its protein identifier (ID) with the EC number allocated by the IUBMB [14]. Table 1 illustrates this paucity of knowledge; it shows the respective distributions per species in both protein databases [11] and pathway databases [12,13] of ID-ECs and their respective medians in the animal, plant and fungal kingdoms. Swiss-Prot displays as many as 335 fungal species containing at least one ID-EC, but their median values are as low as two ID-ECs per species (Table 1). In contrast, only 27 fungal species are included in KEGG (which is restricted to complete published genomes), but their median values are as high as 855 ID-ECs per species (Table 1). This contrast is mainly because the public databases surveyed in Table 1 include data on *S. cerevisiae*, which is among the three best fungal genomes correctly annotated at the enzymatic level (data not shown). Most other fungi have limited or null functional annotation, explaining why the median values are so low in MetaCyc and Swiss-Prot.

**Table 1:** Distribution of ID-EC per kingdom in public databases

	Number of species displaying IDs annotated with EC number (ID-EC)			Median value of the set of ID-EC found per species		
	
	KEGG	MetaCyc	Swiss-Prot	KEGG	MetaCyc	Swiss-Prot
Animal	38	7	1252	2021	2	1
Fungi	27	14	335	855	3	2
Plant	6	158	915	1051	2	1

This remarkable situation arises largely because there is currently no tool for large-scale analyses of fungal metabolism, except for a preliminary attempt to identify enzymes in pathogenic fungi for a limited number of metabolic pathways [15]. To cope with this major shortcoming, we designed a tool that allows us to mine genomic data by combining two complementary approaches: (i) defining reliable groups of orthologous proteins and (ii) mapping these groups on to the metabolic pathways that are described in KEGG [12] and MetaCyc [13].

## Organizing relevant data for analyzing fungal metabolic pathways

### Identifying enzyme activities requires relevant prediction of orthologs

As more and more genomic data become available, homology can be used to reconstruct the metabolic pathways of newly-sequenced organisms, taking the pathways of well-studied model organisms such as yeast as reference. Accordingly, one must identify the amino acid sequences encoding each step of each pathway in organisms that have not been studied experimentally [16-18]. However, there are two major drawbacks in this transfer of information. First, the accuracy of functional annotation of many fungal genomes is low because experimental data are lacking except in the case of yeast [19-21]. Secondly, it is difficult to predict reliable orthologs among all the putative homologs detected during exhaustive comparison of pairs of genomes. Numerous methods have been published but none appears completely infallible (for a recent review, see [22]). Thus, we decided to apply independent methods to the same dataset, collect as many potential orthologs as possible, and then compute their overlap. Exploring several methods raised the probability of finding consistent groups corresponding to this overlap. Accordingly, we used three different and complementary approaches based on similarity searches, and another based on the analysis of phylogenetic trees of families of homologs.

#### Searching pertinent orthologs

First, two published methods were used with their respective default parameters. Inparanoid [8] allows us to identify the orthologs and the inparalogs (genes duplicated since the last speciation event) during pairwise genome comparison. OrthoMCL [9] permits consistent strongly-related groups of orthologs (including inparalogs) to be identified.

Secondly, we improved the classical all-versus-all BLASTP [23] approach to identifying pairs of best reciprocal hits (BRH) [24] with a dedicated Perl script, enhancing the definition of orthologs by specifying two parameters, the alignment percentage and the score ratio, to filter the BLAST results. Local conservation was avoided by dividing the alignment length of each aligned sequence by its total length. The score ratio is defined as the ratio of the raw BLAST score computed by aligning a pair of sequences to the raw score of each sequence against itself (i.e. maximum score). Only results with score ratios over 0.2 and alignment percentages above 60% were kept for further studies.

These different methods based on sequence similarity yield various clusters of orthologous proteins that are more or less stringent depending whether single (e.g. Inparanoid) or multiple (e.g. BRH [Additional File 2]) links are used to build the orthologous protein groups.

Besides these methods based on similarity approaches, methods based on phylogenetic analysis have recently been developed to build orthologous groups [25,26]. Here we chose a phylogenetic approach we had previously developed [25] to obtain groups of orthologous proteins, using automated analysis of trees of families of homologous proteins without a reference tree. The homologous proteins were first detected using BLASTP [23] with the following constraints: an E-value less than 0.001 and an alignment extending for at least 70% of the length of the shorter matching protein. For each family, a multiple alignment was built with Muscle [27], and the phylogenetic tree deduced was reconstructed using PhyML [28]. The program Retree from the Phylip package [29] was further used to root the tree in order to distinguish orthologs from paralogs using automatic tree analysis [25].

Table 2 shows a strikingly low overlap between the results obtained by applying these four methods to the 20 fungal genomes under study. The first column shows that the highest number of groups of orthologs is obtained with the BRH method. However, this may be partly artifactual since BRH is the only method in which an amino acid sequence can belong to different groups owing to the formation of multiple links [Additional File 2]. Columns three to six show the relative percentages shared among the different methods as a double matrix. The upper matrix shows that the relative percentages of identical groups are generally low; the highest figure is 22.4% (common to the OrthoMCL and Inparanoid outputs). The lower matrix shows the percentage of groups that are unique to one of the two methods compared. The low figures obtained (ranging from 8 to 32.6%) suggest that each method brings specific information. The highest specificities are found with the phylogenetic approach, which is indeed the most distinctive of the four approaches we used.

**Table 2:** Groups of orthologous proteins for the 20 genomes available in FUNGIpath predicted by four different methods

Total Number	Relative percentage sharing between two methods				
	
		BRH	Inparanoid	OrthoMCL	Phylogeny
52292	BRH	-	4.8%	3.7%	5.8%
18235	Inparanoid	8.0%	-	22.4%	8.5%
20379	OrthoMCL	12.4%	16.3%	-	8.3%
12676	Phylogeny	32.4%	25.9%	32.6%	-

upper triangular matrix: percentage of identical groups

lower triangular matrix: percentage of specific groups

#### Identifying biologically relevant groups of orthologs

Although the overlap between these different methods for detecting orthologs appears narrow, we tried to build a consensus of the groups of orthologs using both union and intersection methods. Consideration of all the orthologs found merged large numbers of proteins (2,694 proteins in the largest group), with a trend towards amalgamating sometimes quite distant groups of orthologs. On the other hand, computing the crude intersection of the different methods also seemed inadequate (32 proteins in the largest group), since the BRH approach does not detect the inparalogs found by the other methods.

**Figure 1:**
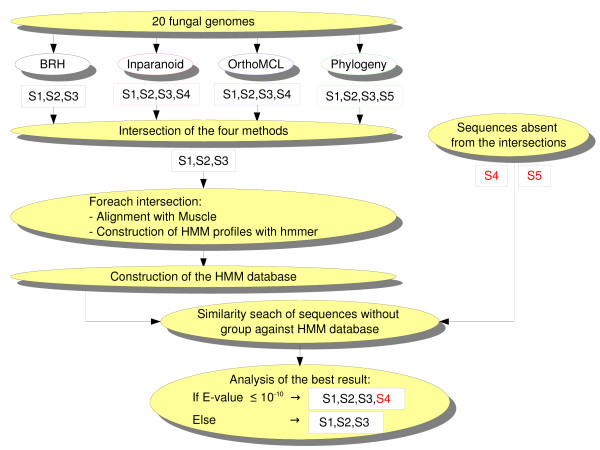
**Flow chart of the construction of orthologous groups**. The diagram represents an overview of our annotation process. We applied four methods of orthologous predictions (BRH, Inparanoid, OrthoMCL and Phylogeny) to 20 fungal genomes. We compared the different groups (represented in the diagram by the sequences S1, S2, S3, S4 and S5) and we constructed their intersections (represented in the diagram by S1, S2 and S3). For each intersection, we aligned the sequences using Muscle [27] and built a HMM profile using HMMER [30]. All these HMM profiles were merged in one file to create a HMM database. Thus, we searched all sequences without a group (S4 and S5 for instance) for shared similarity with one HMM profile (HMMER hmmpfam program) [30]. According to the E-value, we attributed the sequence (E-value ≤ 10^-10^) to the corresponding group or excluded it from that group (E-value > 10^-10^).

To cope with these difficulties, we modified the intersection approach, using a two-step strategy based on enrichment of the reference groups, i.e. the groups of orthologs obtained by the crude intersection approach. Fig. 1 shows a flowchart of our approach. (i) For each reference group, the sequences were aligned [27] and their corresponding HMM profile was computed using the HMMER hmmbuild and hmmcalibrate programs [30]. To avoid any bias due to the numerous inparalogs present in some species, only one homologous gene per genome was conserved as the reference ortholog building the individual HMM profiles. (ii) All the computed HMM profiles were organized as a database, and each sequence not included in any reference group was further compared to the database using the HMMER hmmpfam program [30] in order to add it to a reference group using stringent threshold. Indeed, to build sound *final *groups, we limited the assignment of a sequence to a reference group if the E-value was less than a threshold of 10^-10 ^[Additional File 3]. This stringent criterion allowed a good balance to be kept between sensitivity (29.2% of the sequences initially not associated with a reference group were now associated with one) and specificity (64.2% of the sequences initially found by at least one method but associated with several groups of orthologs were now associated with only one).

In total, we obtained 12,850 final groups of orthologs (size ≥ 2) that appear biologically relevant, the largest group containing 297 sequences (see the size distribution in [Additional File 4]). These figures suggest a good compromise when compared with the values obtained using the crude union and intersection methods (Table 3). With such a prediction, 57% of the total sequences were associated with a group of orthologs [Additional Files 5, 6, and 7]. The mean number of homologous proteins per genome is close to 1.3 in all final groups, suggesting that the orthology/paralogy relationships are quite well resolved by our enrichment approach. Comparison of our ortholog predictions with the four initial methods (Table 4) shows that our approach gives results different from each separate method. The highest number of identical groups with FUNGIpath is obtained with OrthoMCL, whereas the lowest percentage of specific groups is obtained with Inparanoid and BRH.

**Table 3:** Sampling the orthologs in relevant groups

Method	Union	Intersection	HMM profile and enrichment
Total number of groups	12985	12985	12850

Size of the largest group	2694	32	297

**Table 4:** Comparing the orthologous groups predicted by FUNGIpath and by the four methods initially used

	BRH	Inparanoid	OrthoMCL	Phylogeny	Average
Percent of groups identical with FUNGIpath	2.8%	18.6%	18.8%	10.7%	**12.7%**
Percent of groups specific in FUNGIpath	10.6%	10.6%	23.4%	24.6%	**17.3%**

#### Assessing the reliability of the predicted final groups of orthologs

To ascertain the reliability of our predictions further, we computed a confidence score *S *for each final group of orthologous proteins, as follows:

where *m *is the number of methods used for orthology prediction, *I*_*F, i *_is the number of orthologs shared (intersection) between the result of method *i *and the final group of orthologs, O_*F *_is the number of orthologs in the final group and G_*i *_is the number of groups obtained by method *i *for the set of proteins composing the final group. This confidence score is based on the assumption that the reliability of a final group increases with the number of independent methods that find it. Thus, if method *i *predicts the attested group, the score is 1. If not, the score is greater than 0 and less than or equal to 1. The average score (computed as the sum of scores for each method divided by the total number of methods *m*) was scaled from 0-10 by multiplying by 10; the higher the score, the better the agreement among the four methods. With this scoring approach, the user of FUNGIpath can evaluate the reliability of each predicted group of orthologs at any time.

### Reconstructing pathways

#### Transferring EC number annotations to predicted groups of orthologs

Once the final groups of orthologs have been defined and attested, the functional annotations defined for well-studied proteins referenced in reliable public databases can be transferred to homologous unannotated amino acid sequences. For that purpose, an HMM profile was built for each final group of orthologs after multiple alignment of their sequences [27] and use of the HMMER programs (hmmbuild and then hmmcalibrate [30]). We then searched all the HMM profiles against the sequences annotated with a valid four-digit EC number available in Swiss-Prot release 56.7 using the HMMER hmmsearch program [30]). The Swiss-Prot functional annotation was transferred to all members of a group of orthologs displaying a best hit E-value ≤ 10^-80^. The E-value threshold was lowered to 10^-20 ^if at least one sequence of the group of orthologs was already endowed with the same Swiss-Prot annotation.

This approach allows fungal annotation to be improved by using the enzymatic annotation of any protein, irrespective of the phylum in which it was first described. Accordingly, we could transfer 864 EC numbers to 1399 of the 12850 groups of orthologs; if the fungal Swiss-Prot annotations were directly transferred, the number of groups would be only 935. This allowed 160 EC numbers to be added that were not present in fungal genomes in Swiss-Prot [11].

Note that as many as 349 EC numbers (40% of the total of 864) are present in the 20 genomes.

#### Numbering pathways defined by KEGG and/or MetaCyc

Once the different putative orthologs had been annotated as described above, we used them to predicting the different metabolic pathways exhaustively in the completely sequenced fungi under study. To do that, we used two reliable public databases, KEGG [12] and MetaCyc [13], which differ in the way they define pathways.

KEGG [12] defines so-called reference pathways, agglomerating related elementary pathways, while MetaCyc [13] is a universal metabolic database that presents the elementary pathways encoded by various organisms (1,500) separately, including variants (similar biochemical functions using different biochemical routes or similar sets of reactions). KEGG [12] was used to extract useful information from the reaction file and to download all corresponding GIF maps. BIOPAX (BIOlogical PAthway eXchange) files defined in MetaCyc [13] were downloaded and we automatically generated map pictures by directed graph building. We thus collected 154 reference pathways in KEGG and 1386 elementary pathways in MetaCyc, which define the main anabolic and catabolic routes.

#### Challenging the FUNGIpath predictions

To test the soundness of the data computed in FUNGIpath, we compared the predictions made for the model organism *S. cerevisiae *with the information published for the same ID-EC in four curated public databases: Swiss-Prot (release 56.7) [11], KEGG (version 2009-02-02) [12], MetaCyc (release 12.5) [13], and SGD (version 2009-02-10) [31]. Table 5 compares each database against the four others. Each public database appears to have its own specificity and the overlaps between pairs of the databases compared are significantly low, especially in respect of the large differences between the total numbers of ID-ECs (e.g. 1,101 in KEGG versus 527 in SGD). Table 5 also shows that the percentage of ID-ECs that are identical between public databases is at best 60% (KEGG versus Swiss-Prot). Although we mainly used Swiss-Prot data to predict enzymatic annotation in FUNGIpath, the relative percentage of identical ID-ECs was only 68%: 16% of Swiss-Prot annotations were not confirmed by the experimental strategy we used to build FUNGIpath, while 16% of FUNGIpath predictions were absent from Swiss-Prot.

**Table 5:** Comparing the *S. cerevisiae *enzymatic data published in four different databases with those predicted in FUNGIpath

				Distribution of ID-EC (percentage of larger database content)		
				
Database 1	Total ID-EC	Database 2	Total ID-EC	Identical	Specific to database 1	Specific to database 2
KEGG	1101	MetaCyc	155	127 (11%)	974 (86%)	28 (2%)
KEGG	1101	SGD	527	409 (34%)	692 (57%)	118 (10%)
KEGG	1101	Swiss-Prot	1261	889 (60%)	212 (14%)	372 (25%)
**KEGG**	**1101**	**FUNGIpath**	**1261**	**844 (56%)**	**417 (27%)**	**257 (17%)**
MetaCyc	155	SGD	527	132 (24%)	23 (4%)	395 (72%)
MetaCyc	155	Swiss-Prot	1261	136 (11%)	19 (1%)	1125 (88%)
**MetaCyc**	**155**	**FUNGIpath**	**1261**	**134 (10%)**	**21 (2%)**	**1127 (88%)**
SGD	527	Swiss-Prot	1261	433 (32%)	94 (7%)	828 (61%)
**SGD**	**527**	**FUNGIpath**	**1261**	**419 (31%)**	**842 (62%)**	**108 (8%)**
**FUNGIpath**	**1261**	**Swiss-Prot**	**1261**	**1024 (68%)**	**237 (16%)**	**237 (16%)**

To understand these differences better, we looked more closely at the similarities of EC numbers between FUNGIpath and the four public databases. Table 6 shows the distribution of identities at each digit of the shared EC numbers. It appears that the FUNGIpath predictions correspond to more than 80% of the EC numbers found in the other databases. In addition, it can be seen that almost all the differences are limited to the fourth digit, corresponding mainly to the nature of the substrate of the enzyme compared. If we compare our predictions with a predictor such as PRIAM [32], we note that 50.9% of the enzymatic annotations are identical and only 6.8% are different (the difference being mainly in the last EC number digit). The remaining 42.3% are specific to PRIAM (18.6%) or FUNGIpath (23.7%). Thus, the reliability of the automatic approach used by FUNGIpath, predicting groups of orthologous proteins and annotating their enzymatic function, seems comparable with that of other tools or the independently curated public databases. This is true whether the functional annotation is based mainly on experimental data (e.g. SGD) or on sequence similarity (e.g. KEGG).

**Table 6:** Comparing the *S. cerevisiae *enzymatic data predicted in FUNGIpath with public databases

		Number of ID-EC in FUNGIpath		Number of differences at digit position			
			
Public Database	Total ID-EC in FUNGIpath	identical	different	1^st^	2^nd^	3^rd^	4^th^
KEGG	1012	844 (83.4%)	34 (3.4%)	1	1	5	27
MetaCyc	148	134 (90.5%)	8 (5.4%)	2	0	1	5
SGD	504	419 (83.1%)	32 (6.3%)	5	2	3	22
Swiss-Prot	1055	1024 (97.1%)	27 (2.6%)	1	0	3	23

**Figure 2:**
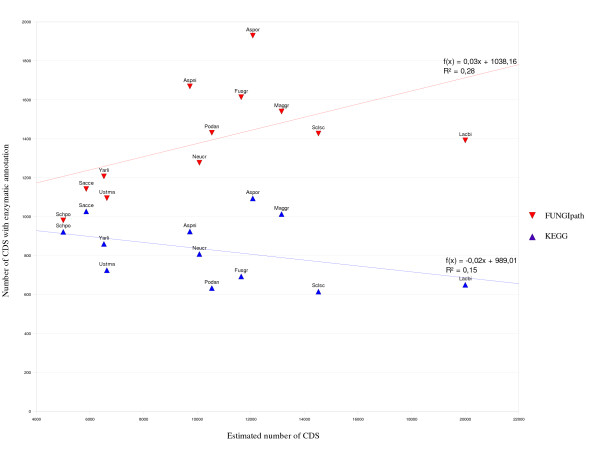
**Correlation between genome size and number of proteins annotated with a four-digit EC number**. The x-axis represents the genome size computed as the estimated number of CDS and the y-axis is the number of CDS endowed with enzymatic annotation. The red triangles correspond to the FUNGIpath data and the blue triangles to the KEGG data.

**Figure 3:**
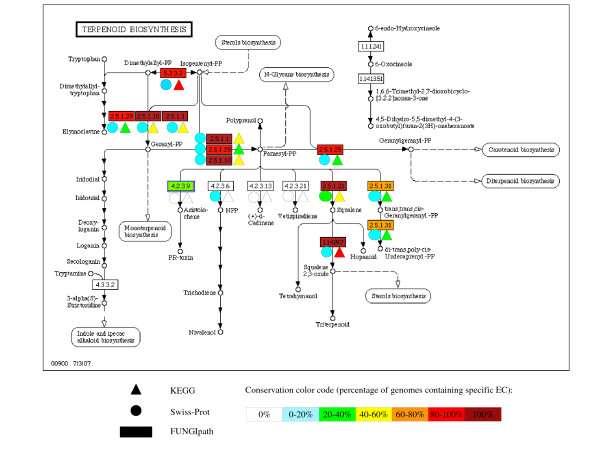
**Comparison of the levels of conservation of the 'terpenoid biosynthesis' pathway according to different sources (Swiss-Prot, KEGG and FUNGIpath)**. The level of conservation of each EC number involved in the 'terpenoid biosynthesis' pathway was computed in FUNGIpath (rectangle) and two public sources. The coloured triangles represent the Swiss-Prot data for the 17 species shared with FUNGIpath. The coloured circles stand for the KEGG data for the 12 species shared with FUNGIpath.

Moreover, Table 7 shows the level of agreement when functional annotations for 12 species established by KEGG [12] and FUNGIpath are compared. Strikingly, the average number of specific ID-EC predictions is larger in FUNGIpath (1,551) than in KEGG (879) and their distribution is unexpected. Only 647 (38%) are strictly identical and 30 more are nearly identical, mostly differing only at the level of the last EC number digit, suggesting that we predicted the right reaction but the substrate is uncertain [Additional File 8]. Four times more predictions are specific to FUNGIpath (48%) than to KEGG (12%). This result is probably not due to any overprediction effect. Indeed, many enzyme predictions have been curated manually in *S. cerevisiae *and in this case the results are fairly close (15% for KEGG against 26% for FUNGIpath). Moreover, the corresponding figures for Swiss-Prot and FUNGIpath are 17.6% and 13.7%, respectively [Additional Files 9, 10]. To check whether there is any correlation, we plotted the genome size and the number of sequences with enzymatic annotations predicted respectively by FUNGIpath and KEGG (Fig. 2). We obtained a better correlation for the FUNGIpath data (R^2 ^= 0.28), and the slope of the tendency curve was positive with the FUNGIpath predictions but negative with the KEGG predictions. Thus, there seems to be no strong methodological bias that could explain why the predictions of FUNGIpath are generally far better than those of KEGG and close to those of the well-curated Swiss-Prot database. In fact, we observed that a significant number of the Swiss-Prot-specific IDs have no orthologs in other genomes, explaining why they are not detected in FUNGIpath. Thus, the high number of specific FUNGIpath predictions obtained is probably due to neither under-prediction by KEGG nor over-representation by FUNGIpath. Indeed, the average numbers of proteins that are annotated for an enzymatic reaction in KEGG and FUNGIpath are quite close (respectively 9.5 and 13.5% [Additional File 11]). The main reason for the better performance of FUNGIpath is probably our choice to work only with complete EC numbers [33], allowing a significant portion of the incomplete KEGG EC numbers to be recovered. For instance, 92 (25%) of the 388 incomplete EC numbers in KEGG have been completed in FUNGIpath. This enrichment by FUNGIpath is illustrated by comparing the information given by the different databases for the KEGG reference pathway 'terpenoid biosynthesis' (Fig. 3). When the level of pathway conservation is compared among the FUNGIpath, KEGG and Swiss-Prot predictions, we observe that this level is globally lowest with the Swiss-Prot data and higher in KEGG, but the highest conservation is obtained with FUNGIpath. These differences can be explained by the better annotation of fungal genomes in FUNGIpath.

**Table 7:** Comparison of enzymatic data between KEGG and FUNGIpath based on the 12 species they share

	Number of ID-EC		Number of			
		
Genome	KEGG	FUNGIpath	Identical ID-EC	Same ID with different EC	KEGG specific ID-EC	FUNGIpath specific ID-EC
*Aspergillus nidulans*	967	1890	675 (31%)	30 (1%)	262 (12%)	1185 (55%)
*Aspergillus oryzae*	1142	2148	853 (36%)	45 (2%)	244 (10%)	1250 (52%)
*Fusarium graminearum*	725	1786	535 (27%)	26 (1%)	164 (1%)	1225 (63%)
*Laccaria bicolor*	684	1536	472 (27%)	31 (2%)	181 (11%))	1033 (60%)
*Magnaporthe grisea*	1070	1801	749 (36%)	39 (2%)	282 (14%))	1013 (49%)
*Neurospora crassa*	852	1407	658 (42%)	26 (2%)	168 (11%)	723 (46%)
*Podospora anserina*	665	1594	473 (27%)	18 (1%)	174 (10%)	1103 (62%)
*Saccharomyces cerevisiae*	1101	1261	844 (57%)	35 (2%)	222 (15%)	382 (26%)
*Schizosaccharomyces pombe*	1009	1073	752 (58%)	33 (3%)	224 (17%)	288 (22%)
*Sclerotinia sclerotiorum*	651	1601	493 (28%)	16 (1%)	142 (8%)	1092 (63%)
*Ustilago maydis*	772	1206	546 (39%)	35 (3%)	191 (3%)	625 (45%)
*Yarrowia lipolytica*	909	1311	710 (48%)	27 (2%)	172 (12%)	574 (39%)

Average	879	1551	647 (38%)	30 (2%)	202 (12%)	874 (48%)

## Using FUNGIpath

FUNGIpath http://www.fungipath.u-psud.fr has been designed as a user-friendly website. Implemented in PHP, HTML and Javascript, it allows various aspects of fungal cell biology to be studied by performing specific predetermined queries on a PostgreSQL [34] database containing primary (genome sequences, metabolism information) and secondary (orthology) data. The sources of the fungal genomes are indicated in [Additional File 12]. An overview of the database is available in [Additional File 13].

A few examples of the proposed queries are given below.

### Querying orthologs

**Figure 4:**
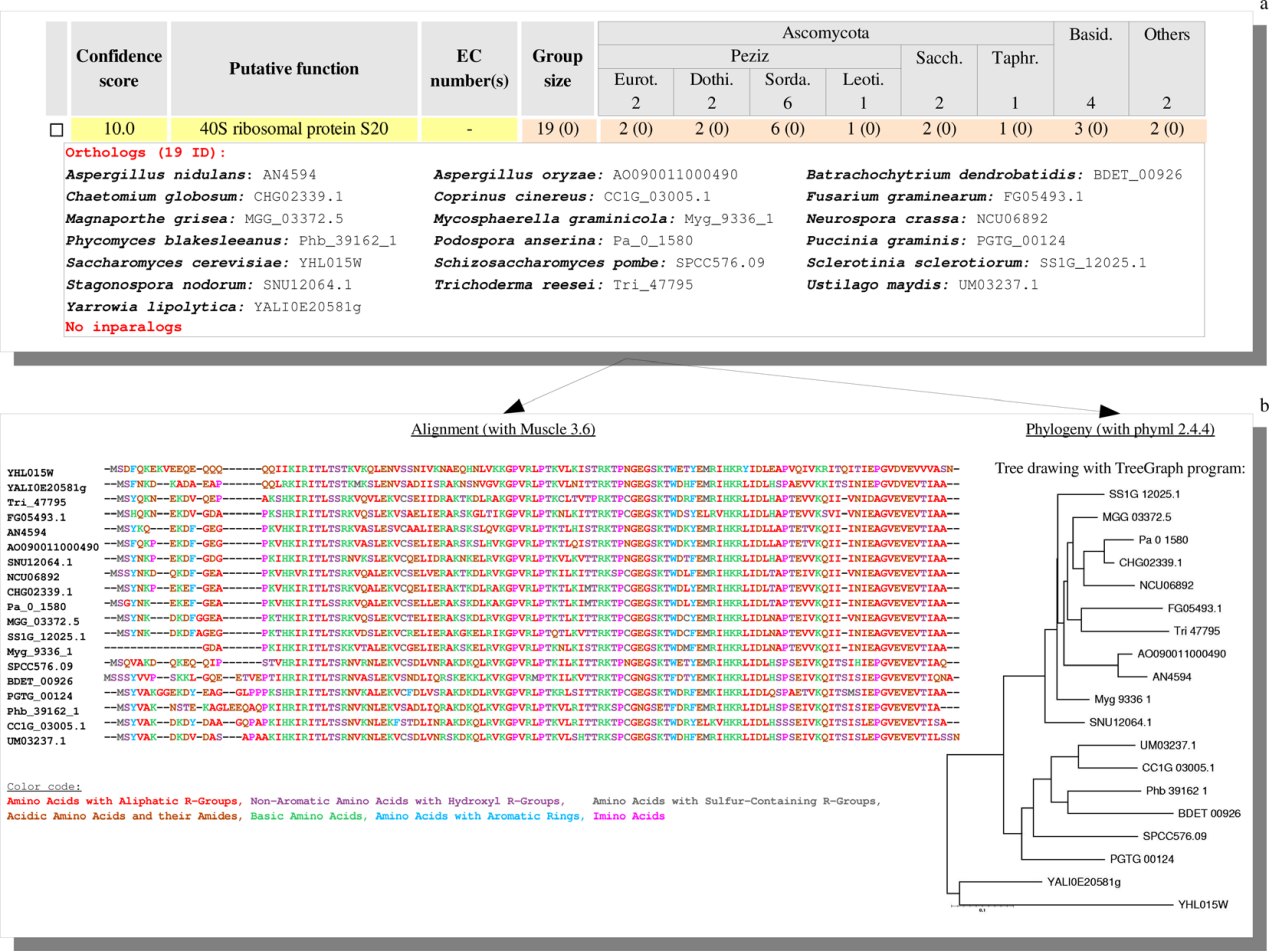
**Searching groups of orthologous proteins**. **a**: Results obtained when searching groups containing the protein ID UM03237.1. The first four columns give general information about the group and the later columns indicate the profile of conservation for the different groups of fungi. Putting the mouse on the group size displays a tooltip with its IDs and their species names. **b**: Alignment [27] and phylogeny [28] of the 20 sequences of this group of orthologs are immediately available by clicking on the group size.

It is possible to seek out orthologs present in the full set of genomes or to restrict queries on specific subsets defined by taxonomic or other criteria. One can use either a sequence or its sequence identifier (if available). Fig. 4a shows a typical output of such queries. Each resulting group of orthologs is associated with its confidence score (computed as described supra), a putative function (if any), an EC number (if available), the group size and the conservation profile for the previously selected species. The list of orthologous (including inparalog) IDs belonging to the selected species can also be displayed. Moreover, as shown in Fig. 4b, its multiple sequence alignment can be computed in the process, and the topology of its deduced phylogenetic tree can then be examined, in order to evaluate the predicted group and to assess its relevance in terms of range of sequence identity and functional annotation.

For instance, querying the sequence UM03237.1 belonging to the *Ustilago maydis *genome defines a final group that is found whichever method is used (confidence score is maximal) and displays an alignment of quite good quality. Thus, the likelihood of this group of orthologous proteins seems quite reasonable if we combine the high-level quality of the score and the suitability of its alignment.

### Exploring pathways

**Figure 5:**
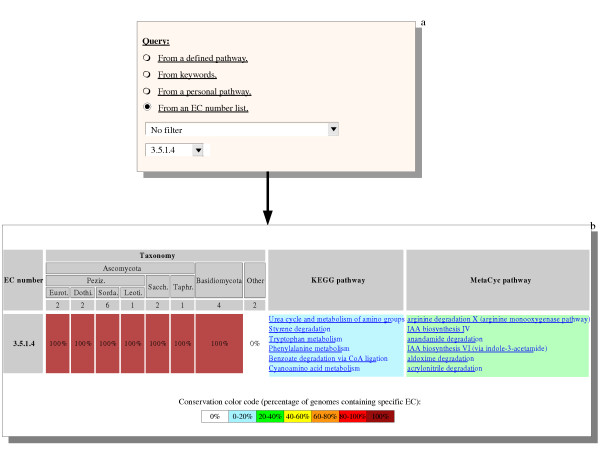
**Exploring pathways using a specific EC number**. **a**: The EC number 3.5.1.4 was searched against all 20 genomes available in the database. **b**: The level of conservation among the different groups belonging to different taxonomic groups of fungi is indicated with a colour code (from white (0%) to dark red (100%)). The pathways that contain the requested EC number are listed in the KEGG and MetaCyc columns, respectively. Note that the pathway names are different in these two databases.

**Figure 6:**
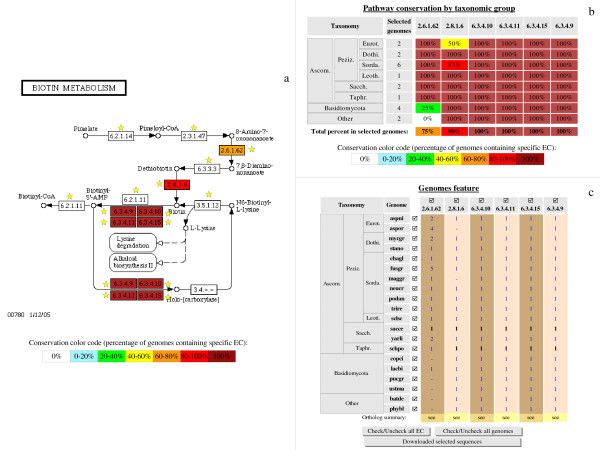
**Analysis of 'biotin metabolism' defined by KEGG**. The 'biotin metabolism' reference pathway was searched against all 20 genomes available in the database. The conservation colour code is the same as in Fig. 5. **a**: The EC numbers specific to this pathway are indicated by a yellow star. **b**: The table lists the percentages of conservation of this pathway in each species forming the different taxonomic groups of fungi. c: The table lists the presence (number of sequences) or absence (dash) of orthologs for each enzyme. The species are sorted by taxonomic group.

FUNGIpath further allows the conservation of pathways between different fungi to be checked and visualized. This can be done either at the level of a particular step (corresponding to a defined EC number) in a pathway or by considering all the steps of a complete pathway. Figs. 5 and 6 detail the different strategies used by FUNGIpath (see below). Moreover, one can handle a user-defined pathway delineated in a simplified BIOPAX format (data not shown).

#### Searching a specific step in a pathway

Searching a specific EC number (Fig. 5a) allows the level of conservation of this enzyme activity in each taxonomic group to be assessed; also the full list of pathways to which this EC number is predicted to belong can be obtained directly (Fig. 5b). For instance, Fig. 5 shows that acylamide amidohydrolase (EC 3.5.1.4) is very well conserved in fungi and is involved in at least six different pathways in both the KEGG and MetaCyc databases (Fig. 5b). Since this activity is used in so many pathways of both primary and secondary metabolisms, it is not surprising to find this EC number in ten distinct groups of orthologous proteins ranging in size from 4 to 25 members (data not shown). The distribution of the different orthologs and inparalogs present in these groups can be further used to study the evolution of these different pathways using the approaches described in Fig. 4.

#### Searching a complete pathway

**Figure 7:**
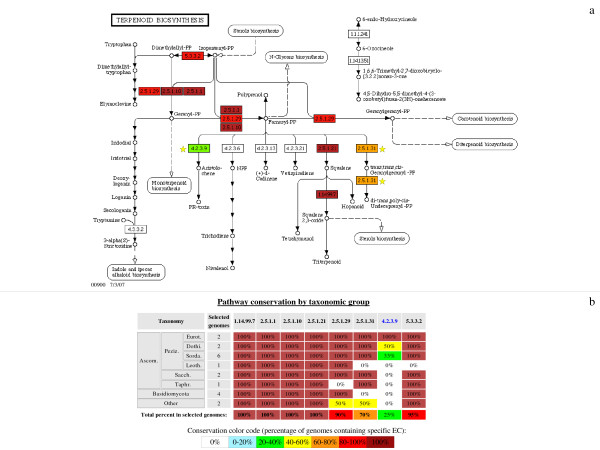
**Exploring pathways using a specific pathway name**. The 'terpenoid biosynthesis' reference pathway defined by KEGG was searched against all 20 genomes available in the database. **a**: Each EC number has been coloured according to its global level of conservation as in Figs. 5 and 6. Two EC numbers (2.5.1.31 and 4.2.3.9) specific to this pathway (indicated by a yellow star) are not detected in all species studied. **b**: This table lists the percentage conservation of each EC number in this pathway among all taxonomic groups of fungi. Its global presence in all taxonomic groups is given in the last line of this table.

It is further possible to assess the level of conservation of each EC number in a complete pathway. Figs. 6 and 7 illustrate the available queries we propose in order to analyze primary (e.g. biotin metabolism) and secondary (e.g. terpenoid biosynthesis) pathways, respectively. The results are presented as both a KEGG gif map (Figs. 6a and 7a) and a table listing the presence/absence of each step in the pathway in the various fungal species (Figs. 6b and 7b), examining the EC numbers associated with each step. The conservation level of the different steps in the pathway is indicated by a colour code from dark red (100%) to white (0%). Groups of orthologous proteins associated with the conserved EC numbers are listed in the genome features table (Fig. 6c). Note that rich information is available and can be viewed using mouse-over facilities on many - 'explicit' and 'implicit' - links; for example, protein sequences can be downloaded for further study.

Fig. 6 shows that only two of the five steps in biotin biosynthesis are highly conserved. EC 2.8.1.6 is detected in all the species compared except *Aspergillus oryzae *and *Magnaporthe grisea*. EC 2.6.1.62 is absent from several species (*Coprinus cinereus*, *Puccinia graminis*, *U. maydis*, *Batrachochytrium dendrobatidis *and *Phycomyces blakesleeanus*). Thus, the KEGG reference pathway 'biotin metabolism' (Fig. 6a) appears to be incomplete in many fungi, since several of its specific enzyme activities (EC 2.3.1.47, 3.5.1.12, 6.2.1.14, 6.2.1.11 and 6.3.3.3) are not found. We may suppose that either these EC numbers exist in the fungi but are not currently detectable, or the fungi use other enzyme activities to catalyse these reactions (see below). Moreover, the further steps in biotinylation catalyzed by the ligases EC 6.3.4.9, 6.3.4.10, 6.3.4.11, and 6.3.4.15 are fully conserved in all the main taxonomic groups of fungi.

Fig. 6c further shows that most of the EC numbers (blue text) correspond to proteins that have no EC number assignment in Swiss-Prot but have been annotated in FUNGIpath by orthology prediction. Only two of the twenty genomes (*S. cerevisiae *and *Schizosaccharomyces pombe*) have an annotation in these two databases (bold text).

Fig. 7 shows that only six of the 13 EC numbers involved in the KEGG reference pathway 'terpenoid biosynthesis' appear to be conserved among the fungi analyzed. Of these six EC numbers, four (EC 1.14.99.7, 2.5.1.1, 2.5.1.10 and 2.5.1.21) are found in all the species present in FUNGIpath. Some EC numbers are missing from only one fungal group: this seems to be the case for EC 5.3.3.2, which is absent in the Taphrinomycotina group. Note, however, that this group is represented by only one species, namely *S. pombe*. Two EC numbers (4.2.3.9 in green and 2.5.1.31 in orange) seem to be specific to certain fungi.

## Discussion

Fungal metabolism is exceptionally rich and complex [35], generating a wide variety of secondary metabolic pathways as these organisms progressively evolved to invade new ecosystems. Except in a few model organisms, very few reactions have been studied experimentally. The present-day facility in obtaining complete genome sequences for organisms that have never been experimentally studied has revealed a wide gap between the knowledge gained by disclosing full repertoires of putative amino acid sequences and ignorance of their actual function.

To close this gap, one needs to transfer functional annotation to putative sequences by homology using inductive instead of hypothetico-deductive approaches (holism versus reductionism) [36]. For metabolism, this allows entire pathways to be reconstructed [37]. In order to facilitate the study of fungal metabolism and its evolution, we have created the tool FUNGIpath, which makes the predictions made on this homology basis publicly available. Thus, it was necessary to design new experimental approaches in order to obtain reliable and sound predictions.

### Collecting reliable orthologs

The first requirement was to detect sound orthologs, knowing that there is no uniquely reliable way to do so [22]. The most commonly-used approach is bidirectional best hits (BRH) of BLAST alignment, with imposition of strict criteria on discriminating E-value over a given alignment length, but various more sophisticated approaches have also been developed [22]. Selecting the best method(s) is not easy. For instance, benchmarking tests suggested that Inparanoid performs best while BRH is good for closely-related species [38]. More recently, BRH was found to give results comparable to the more sophisticated methods [39], but it is limited to finding only a single hit among the multiple possible links between paralogs.

We therefore preferred to use several different approaches simultaneously, three based on sequence similarity and one on phylogeny, to obtain robust results. Since the overlap between the outputs of these four methods is very narrow (a result underlining how conflicting these orthology methods are), we enriched the data found in the intersection of the different methods with a HMM approach. This allowed us to obtain fairly coherent sets of reliable orthologs forming well-defined groups that are of adequate size (the largest containing only 297 sequences) and biologically relevant.

### Using reliable orthologs to improve functional annotation

The second requirement for exploiting these orthology data to predict metabolic pathways in fungal species that have never been studied experimentally was to assign a functional annotation to each group of orthologous proteins. To do that, a correspondence was established between a group and an EC number, defining an enzyme catalyzing a specific step in a known pathway included in the KEGG and MetaCyc databases. Figs. 5 and 6 show how group(s) of orthologs responsible(s) for a specific enzyme can be found and how this EC number is distributed in the different genomes. *Inter alia*, the multiple sequence alignment and the deduced phylogenetic tree can be obtained for each family of orthologs and inparalogs encoding this EC number in the fungi compared. We have provided evidence that FUNGIpath is a reliable tool for annotating enzyme function in an automatically predicted group of orthologous proteins. It gives data that are either comparable to those of the independently curated public databases or, in many cases, better (see Table 6). At any rate, most of the differences appear to be limited to the fourth digit, corresponding mainly to the nature of the substrates of the enzymes compared.

FUNGIpath is also useful for finding the set of orthologs that constitutes an entire pathway. This allows us to determine whether all the steps of the pathway have been predicted and, if so, in how many of the genomes compared that pathway is complete. Indeed, one of the main problems encountered in trying to reconstruct entire pathways from orthology data is the occurrence of missing data [40] such as pathway holes [41]. The absence of an EC number (orphan metabolic activities [42]) may be due to a low percentage identity of the corresponding amino acid sequence or to its replacement with another protein. Alternatively, the simultaneous absence of several EC numbers that belong to a specific pathway would suggest that the entire pathway is absent from the species concerned. This is the case, for instance, in the later steps in the KEGG reference pathway 'terpenoid biosynthesis', where the last three EC numbers are missing (Fig. 7a). However, it is possible that this absence may simply be due to a major annotation problem or to the replacement of this pathway with an alternate, undetected, one.

Overall, FUNGIpath appears to be a useful and innovative tool for helping to resolve some artifactual pathway holes. For instance, it is unique in annotating a group of orthologs found in six species as EC 4.2.3.9 (Fig. 3), aristolochene synthase. No such amino acid sequences are predicted in Swiss-Prot, KEGG or MetaCyc, but the presence of aristolochene synthase has been demonstrated experimentally in two fungi not included in FUNGIpath [43,44], supporting our prediction.

## Conclusions

FUNGIpath appears to be a reliable tool for the analysis of fungal metabolism. It will be especially useful for annotating newly-sequenced genomes of poorly-studied organisms.

Moreover, it allows the respective metabolisms of various taxa to be compared easily. For instance, 101 EC numbers are found uniquely in ascomycetes (data not shown) and may help to delineate the metabolic specificities of the last common ancestor of this group.

As more and more genomes are expected to be decrypted in the near future, tools such as FUNGIpath will be very useful for the progressive reconstruction of primary and secondary metabolisms in the ancestors of the main branches of the present-day fungal tree and for elucidating the evolution of various specific derived metabolisms. FUNGIpath will be updated regularly (at least twice a year) with newly published fungal genomes.

## Availability and requirements

The database is available at http://www.fungipath.u-psud.fr. This web site is optimized for Firefox 2.x and has been successfully tested for Safari 2.0.3 and Internet Explorer 7.0.

## Lists of abbreviations

BRH: Best reciprocal hits; CDS: Coding sequences; EC number: Enzyme Commission number; ID-EC: a unique protein identifier (ID) and EC number pair.

## Authors' contributions

SG built the database and developed the website. OL supervised the work and tested the tool. All authors (SG, BL, and OL) drafted, read and approved the final manuscript.

## Supplementary Material

Additional file 1 **Number of sequencing projects by kingdom**. The table shows the number of published and ongoing genomes for the three kingdoms. Click here for file

Additional file 2 **Example of ID that may be present in several groups determined by the BRH method**. BRH pairs define multiple links between the different orthologous proteins (A) as indicated by bi-directional arrows. Accordingly, the lack of BRH link between proteins A_2 _and A_4_, leads to building two different groups of orthologs. Click here for file

Additional file 3 **Influence of the E-value threshold on the association of a sequence and an HMM profile**. The first table shows the sequence distribution after comparison with the HMM profile database according to the number of methods that initially assign a protein sequence (ID) to an group of orthologs. The second table shows the same results for different E-value thresholds. Click here for file

Additional file 4 **Distribution of orthologous group sizes**. The graph represents the distribution of the group size with the number of sequences in a group on the x-axis and the number of groups on the y-axis. Click here for file

Additional file 5 **Number of sequences per genome**. The table shows, for each genome, the total numbers (and their percentages) of protein sequences, of proteins belonging to groups of orthologs and of proteins endowed with an enzymatic activity (annotated with a EC number). Click here for file

Additional file 6 **Comparison of the percentages of annotated sequences for the 20 fungal genomes**. The graph represents, for each genome, the genome size on the x-axis and the percentage of annotated CDS on the y-axis. Genome abbreviations: AspNi for *A. nidulans*, AspOr for *A. oryzae*, BatDe for *B. dendrobatidis*, ChaGl for *C. globosum*, CopCi for *C. cinereus*, FusGr for *F. graminearum*, LacBi for *L. bicolor*, MagGr for *M. grisea*, MycGr for *M. graminicola*, NeuCr for *N. crassa*, PhyBl for *P. blakesleeanus*, PodAn for *P. anserina*, PucGr for *P. graminis*, SacCe for *S. cerevisiae*, SchPo for *S. pombe*, SclSc for *S. sclerotiorum*, StaNo for *S. nodorum*, TriRe for *T. reesei*, UstMa for *U. maydis *and YarLi for *Y. lipolytica*. Click here for file

Additional file 7 **Distribution of sequence lengths**. The graph represents the distribution of sequence lengths (x-axis) with the number of sequences (y-axis). The red point corresponds to all the sequences and the blue point to the sequences assigned to an orthologous group. Click here for file

Additional file 8 **Analysis of the different enzymatic annotations in KEGG**. The table provides, for each genome, the numbers of ID-EC that diverge and the positions that differ between KEGG and FUNGIpath. Click here for file

Additional file 9 **Comparison of enzymatic data between Swiss-Prot and FUNGIpath (based on 17 shared species)**. The table provides, for each genome, the numbers of ID-EC that are common, divergent or specific between Swiss-Prot and FUNGIpath. Click here for file

Additional file 10 **Analysis of the different enzymatic annotations in Swiss-Prot**. The table provides, for each genome, the numbers of ID-EC that diverge and the positions that differ. Click here for file

Additional file 11 **Comparison of data for FUNGIpath genomes**. The table provides, for each genome, the number of sequences with complete enzymatic annotation for FUNGIpath, KEGG and Swiss-Prot. Click here for file

Additional file 12 **Sources of the genomic data used in FUNGIpath**. The respective sequencing centers of each fungal genome are indicated by the url we used to download the primary genomic data. Click here for file

Additional file 13 **Database schema**. The various tables (schematized as rectangles) are coloured in red (genomic data), yellow (predictions of orthologs), and blue (pathway data). Links between tables are indicated by lines. Foreign key names are displayed in italics. Click here for file
