# Supplementary material for: FUNGIpath: a tool to assess fungal metabolic pathways predicted by orthology
Source: BMC Genomics. 2010 Feb 1;11:81. doi: 10.1186/1471-2164-11-81 (PMC2829015; doi:10.1186/1471-2164-11-81)
Supplement: Additional file 1 — Number of sequencing projects by kingdom. The table shows the number of published and ongoing genomes for the three kingdoms. [file 1471-2164-11-81-S1.PDF]

Kingdom	Genome size (in Mb)	Published genomes		Ongoing genomes	
		Total project	Total species	Total project	Total species
Animals	726	39	35	244	192
Fungi	24	26	26	285	113
Plants	312	11	10	85	59
