# Supplementary figures and images for: FUNGIpath: a tool to assess fungal metabolic pathways predicted by orthology
Source: BMC Genomics. 2010 Feb 1;11:81. doi: 10.1186/1471-2164-11-81 (PMC2829015; doi:10.1186/1471-2164-11-81)

BRH pairs

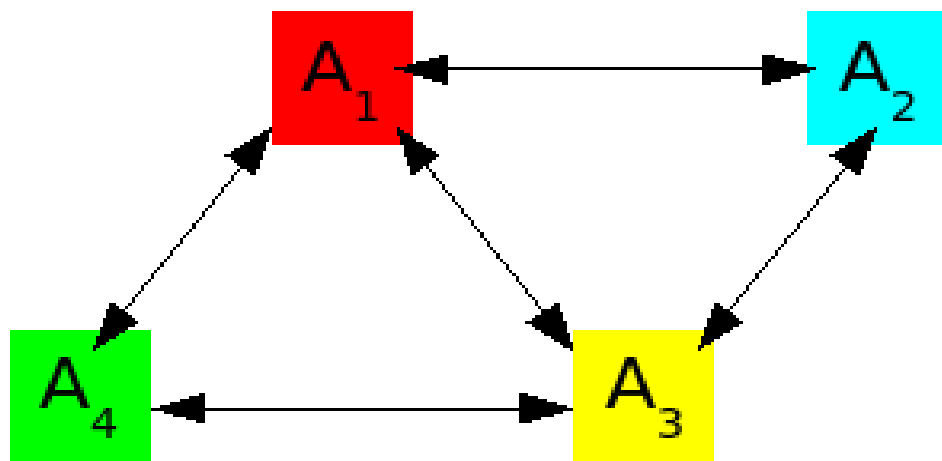

Orthologous groups

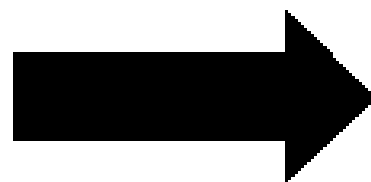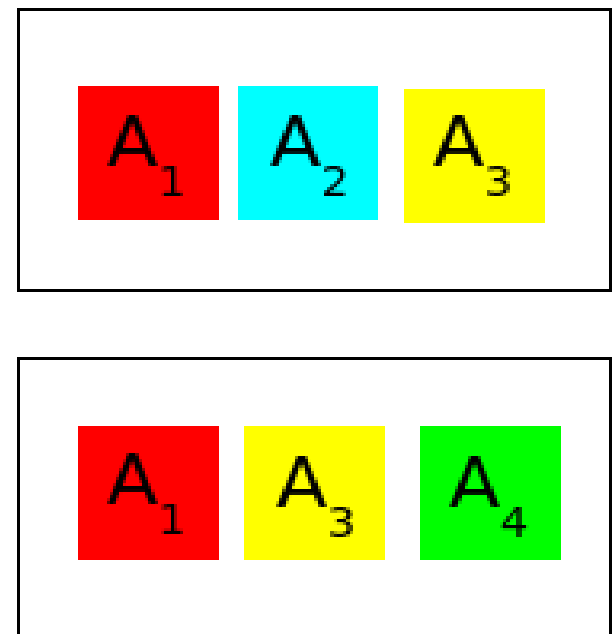

Supplement: Additional file 2 — Example of ID that may be present in several groups determined by the BRH method. BRH pairs define multiple links between the different orthologous proteins (A) as indicated by bi-directional arrows. Accordingly, the lack of BRH link between proteins A2 and A4, leads to building two different groups of orthologs. [file 1471-2164-11-81-S2.PDF]

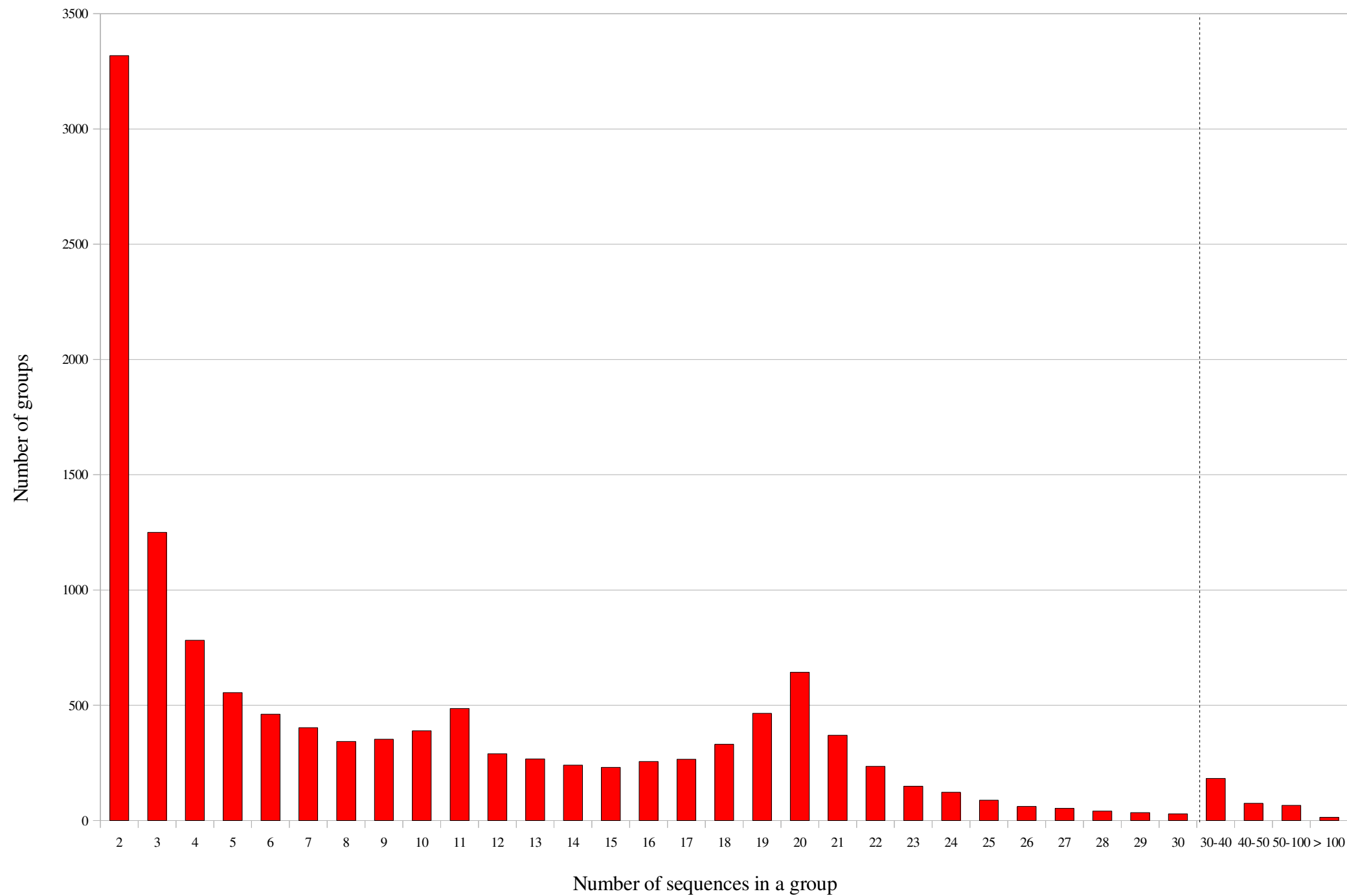

Supplement: Additional file 4 — Distribution of orthologous group sizes. The graph represents the distribution of the group size with the number of sequences in a group on the x-axis and the number of groups on the y-axis. [file 1471-2164-11-81-S4.PDF]

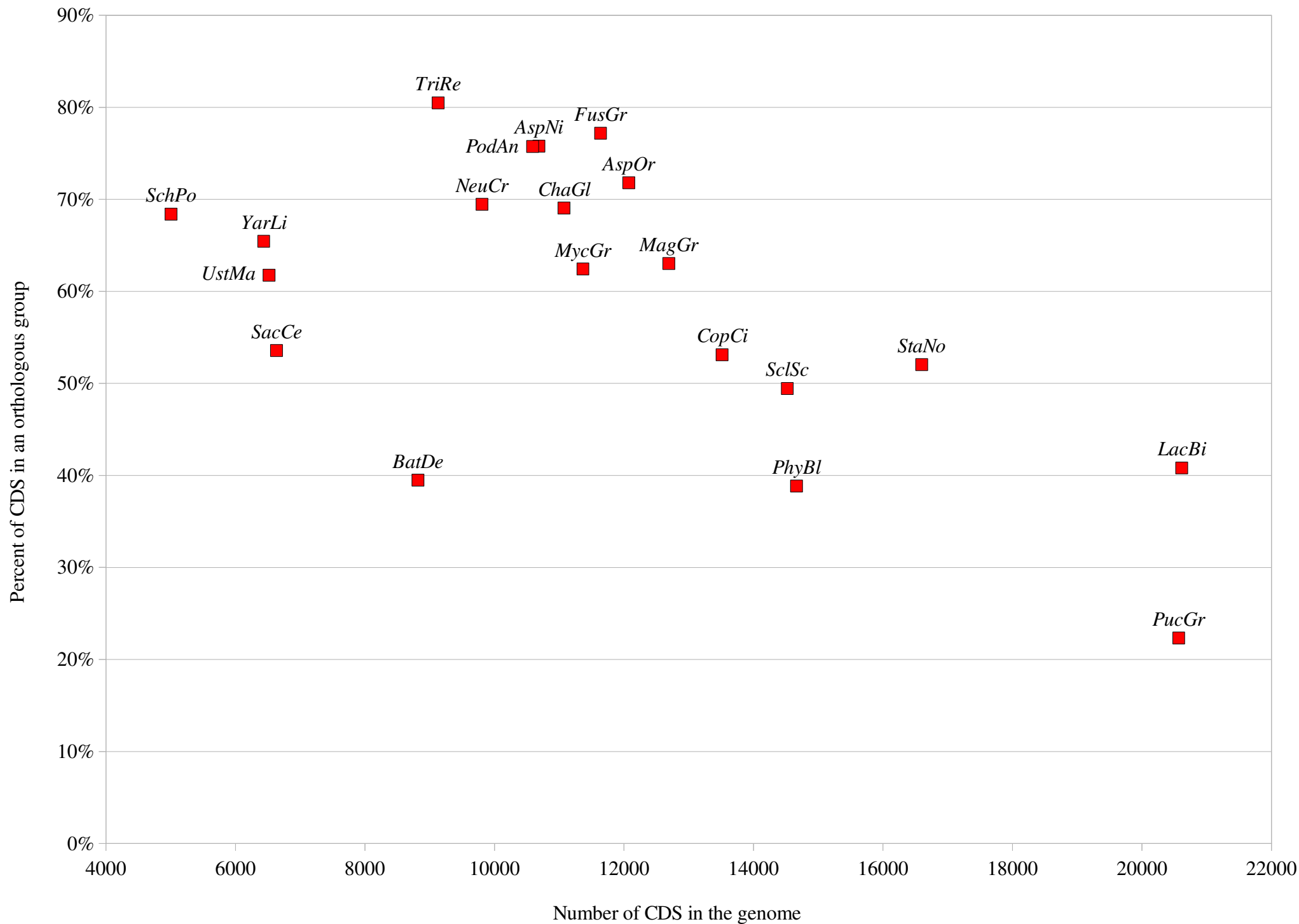

Supplement: Additional file 6 — Comparison of the percentages of annotated sequences for the 20 fungal genomes. The graph represents, for each genome, the genome size on the x-axis and the percentage of annotated CDS on the y-axis. Genome abbreviations: AspNi for A. nidulans, AspOr for A. oryzae, BatDe for B. dendrobatidis, ChaGl for C. globosum, CopCi for C. cinereus, FusGr for F. graminearum, LacBi for L. bicolor, MagGr for M. grisea, MycGr for M. graminicola, NeuCr for N. crassa, PhyBl for P. blakesleeanus, PodAn for P. anserina, PucGr for P. graminis, SacCe for S. cerevisiae, SchPo for S. pombe, SclSc for S. sclerotiorum, StaNo for S. nodorum, TriRe for T. reesei, UstMa for U. maydis and YarLi for Y. lipolytica. [file 1471-2164-11-81-S6.PDF]

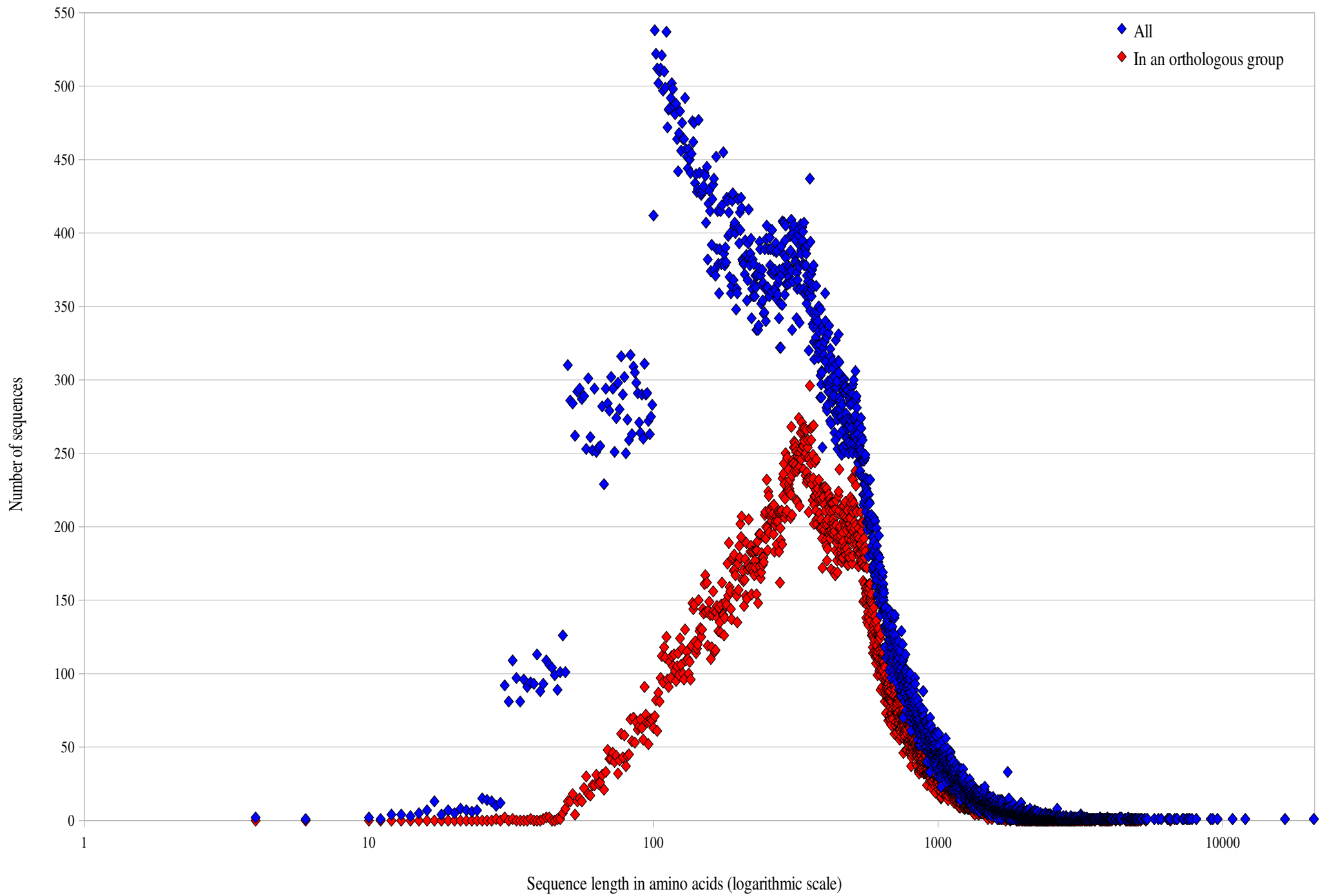

Supplement: Additional file 7 — Distribution of sequence lengths. The graph represents the distribution of sequence lengths (x-axis) with the number of sequences (y-axis). The red point corresponds to all the sequences and the blue point to the sequences assigned to an orthologous group. [file 1471-2164-11-81-S7.PDF]

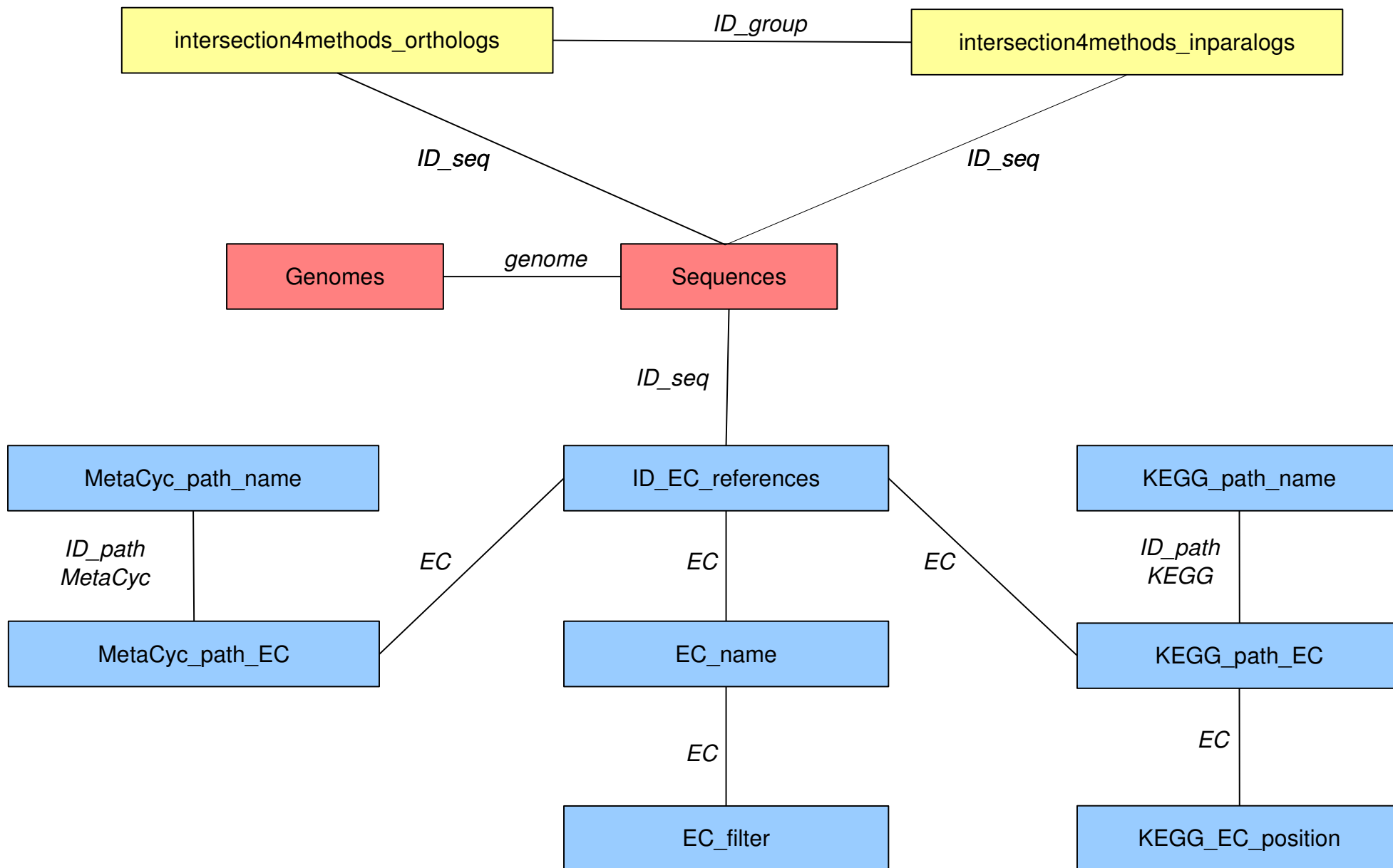

Supplement: Additional file 13 — Database schema. The various tables (schematized as rectangles) are coloured in red (genomic data), yellow (predictions of orthologs), and blue (pathway data). Links between tables are indicated by lines. Foreign key names are displayed in italics. [file 1471-2164-11-81-S13.PDF]
