# Supplementary material for: FUNGIpath: a tool to assess fungal metabolic pathways predicted by orthology
Source: BMC Genomics. 2010 Feb 1;11:81. doi: 10.1186/1471-2164-11-81 (PMC2829015; doi:10.1186/1471-2164-11-81)
Supplement: Additional file 3 — Influence of the E-value threshold on the association of a sequence and an HMM profile. The first table shows the sequence distribution after comparison with the HMM profile database according to the number of methods that initially assign a protein sequence (ID) to an group of orthologs. The second table shows the same results for different E-value thresholds. [file 1471-2164-11-81-S3.PDF]

No result	Number of methods with HMM/ID					Total
	0	1	2	3	4	
40595<br>25%	72663<br>45%	10461<br>7%	20629<br>13%	12551<br>8%	3334<br>2%	160233

E-value threshold	Number of methods with HMM/ID				
	0	1	2	3	4
E-value < 1e-1	83.5%	97.7%	99.1%	99.9%	100%
E-value < 1e-2	82.1%	96.4%	98.6%	99.8%	100%
E-value < 1e-3	59.6%	93.6%	97.1%	99.4%	100%
E-value < 1e-4	47.3%	89.3%	94.8%	98.7%	100%
E-value < 1e-5	41.9%	84.2%	92.4%	97.8%	99.9%
E-value < 1e-10	29.2%	64.2%	77.2%	90.2%	98.9%
E-value < 1e-15	22.8%	55.0%	65.2%	79.9%	97.2%
E-value < 1e-20	19.4%	50.0%	58.1%	72.5%	95.1%
E-value < 1e-25	17.4%	47.0%	54.0%	67.8%	92.8%
E-value < 1e-30	16.1%	45.0%	51.2%	65.2%	90.8%
E-value < 1e-35	15.0%	43.5%	49.0%	62.9%	88.5%
E-value < 1e-40	14.0%	42.1%	47.0%	60.4%	86.3%
E-value < 1e-45	13.1%	40.8%	45.1%	58.0%	84.2%
E-value < 1e-50	12.4%	39.7%	43.5%	56.5%	82.2%
E-value < 1e-55	11.5%	37.9%	41.4%	53.8%	79.6%
E-value < 1e-60	10.9%	36.7%	39.6%	51.8%	77.1%
E-value < 1e-65	10.2%	35.4%	37.8%	49.9%	75.1%
E-value < 1e-70	9.6%	34.1%	36.3%	48.3%	72.9%
