# Supplementary material for: FUNGIpath: a tool to assess fungal metabolic pathways predicted by orthology
Source: BMC Genomics. 2010 Feb 1;11:81. doi: 10.1186/1471-2164-11-81 (PMC2829015; doi:10.1186/1471-2164-11-81)
Supplement: Additional file 5 — Number of sequences per genome. The table shows, for each genome, the total numbers (and their percentages) of protein sequences, of proteins belonging to groups of orthologs and of proteins endowed with an enzymatic activity (annotated with a EC number). [file 1471-2164-11-81-S5.PDF]

Genome	Number of sequences			Percent of sequences	
	All	In orthologous group	Wih EC	In orthologous group	Wih EC
Aspergillus nidulans	10685	8098	1669	75.8%	15.6%
Aspergillus oryzae	12074	8669	1931	71.8%	16.0%
Batrachochytrium dendrobatidis	8818	3482	1005	39.5%	11.4%
Chaetomium globosum	11074	7648	1397	69.1%	12.6%
Coprinus cinereus	13514	7178	1405	53.1%	10.4%
Fusarium graminearum	11638	8983	1614	77.2%	13.9%
Laccaria bicolor	20614	8412	1397	40.8%	6.8%
Magnaporthe grisea	12693	8000	1540	63.0%	12.1%
Mycosphaerella graminicolla	11365	7096	1513	62.4%	13.3%
Neurospora crassa	9807	6812	1277	69.5%	13.0%
Phycomyces blakesleeanus	14664	5696	1619	38.8%	11.0%
Podospora anserina	10589	8021	1431	75.7%	13.5%
Puccinia graminis	20567	4593	1153	22.3%	5.6%
Saccharomyces cerevisiae	6634	3554	1142	53.6%	17.2%
Schizosaccharomyces pombe	5004	3422	983	68.4%	19.6%
Sclerotinia sclerotiorum	14522	7181	1434	49.4%	9.9%
Stagonospora nodorum	16597	8637	1630	52.0%	9.8%
Trichoderma reesei	9129	7347	1395	80.5%	15.3%
Ustilago maydis	6518	4025	1095	61.8%	16.8%
Yarrowia lipolytica	6436	4212	1207	65.4%	18.8%
Total	232942	131066	27837	56,3%	12,0%
