# Supplementary material for: FUNGIpath: a tool to assess fungal metabolic pathways predicted by orthology
Source: BMC Genomics. 2010 Feb 1;11:81. doi: 10.1186/1471-2164-11-81 (PMC2829015; doi:10.1186/1471-2164-11-81)
Supplement: Additional file 8 — Analysis of the different enzymatic annotations in KEGG. The table provides, for each genome, the numbers of ID-EC that diverge and the positions that differ between KEGG and FUNGIpath. [file 1471-2164-11-81-S8.PDF]

Genome	Number of ID-EC different	Digit position which is different			
		1st d.	2nd d.	3rd d.	4th d.
Aspergillus nidulans	30	1	1	3	25
Aspergillus oryzae	45	3	3	4	35
Fusarium graminearum	26	2	1	3	20
Laccaria bicolor	31	1	3	5	22
Magnaporthe grisea	39	3	3	5	28
Neurospora crassa	26	0	2	4	20
Podospora anserina	18	3	0	3	12
Saccharomyces cerevisiae	35	1	1	5	28
Schizosaccharomyces pombe	33	2	3	4	24
Sclerotinia sclerotiorum	16	0	1	3	12
Ustilago maydis	35	1	1	5	28
Yarrowia lipolytica	27	0	2	3	22
