# Supplementary material for: FUNGIpath: a tool to assess fungal metabolic pathways predicted by orthology
Source: BMC Genomics. 2010 Feb 1;11:81. doi: 10.1186/1471-2164-11-81 (PMC2829015; doi:10.1186/1471-2164-11-81)
Supplement: Additional file 9 — Comparison of enzymatic data between Swiss-Prot and FUNGIpath (based on 17 shared species). The table provides, for each genome, the numbers of ID-EC that are common, divergent or specific between Swiss-Prot and FUNGIpath. [file 1471-2164-11-81-S9.PDF]

Genome	Number of ID-EC		Number of				Percent			
	Swiss-Prot	FUNGIpath	Identical ID-EC	Same ID with different EC	Swiss-Prot specific ID-EC	FUNGIpath specific ID-EC	Identical ID-EC	Same ID with different EC	Swiss-Prot specific ID-EC	FUNGIpath specific ID-EC
Aspergillus nidulans	162	1890	143	1	18	1746	7.5%	0.1%	0.9%	91.5%
Aspergillus oryzae	86	2148	79	0	7	2069	3.7%	0.0%	0.3%	96.0%
Chaetomium globosum	26	1580	24	0	2	1556	1.5%	0.0%	0.1%	98.4%
Coprinopsis cinerea	12	1713	6	0	6	1707	0.3%	0.0%	0.3%	99.3%
Fusarium graminearum	42	1786	37	0	5	1749	2.1%	0.0%	0.3%	97.7%
Laccaria bicolor	3	1536	3	0	0	1533	0.2%	0.0%	0.0%	99.8%
Magnaporthe grisea	34	1801	27	0	7	1774	1.5%	0.0%	0.4%	98.1%
Neurospora crassa	235	1407	202	0	33	1205	14.0%	0.0%	2.3%	83.7%
Phycomyces blakesleeanus	10	1762	9	0	1	1753	0.5%	0.0%	0.1%	99.4%
Podospora anserina	23	1594	19	0	4	1575	1.2%	0.0%	0.3%	98.6%
Saccharomyces cerevisiae	1320	1261	1024	27	269	210	67.0%	1.8%	17.6%	13.7%
Schizosaccharomyces pombe	931	1073	842	16	73	215	73.5%	1.4%	6.4%	18.8%
Sclerotinia sclerotiorum	10	1601	7	0	3	1594	0.4%	0.0%	0.2%	99.4%
Stagonospora nodorum	27	1806	25	0	2	1781	1.4%	0.0%	0.1%	98.5%
Trichoderma reesei	21	1551	16	0	5	1535	1.0%	0.0%	0.3%	98.7%
Ustilago maydis	75	1206	55	0	20	1151	4.5%	0.0%	1.6%	93.9%
Yarrowia lipolytica	139	1311	118	2	19	1191	8.9%	0.2%	1.4%	89.5%
Average	186	1590	155	3	28	1432	11.1%	0.2%	1.9%	86.8%
