# Supplementary material for: FUNGIpath: a tool to assess fungal metabolic pathways predicted by orthology
Source: BMC Genomics. 2010 Feb 1;11:81. doi: 10.1186/1471-2164-11-81 (PMC2829015; doi:10.1186/1471-2164-11-81)
Supplement: Additional file 10 — Analysis of the different enzymatic annotations in Swiss-Prot. The table provides, for each genome, the numbers of ID-EC that diverge and the positions that differ. [file 1471-2164-11-81-S10.PDF]

Genome	Number of ID-EC different	Digit position which is different			
		1st d.	2nd d.	3rd d.	4th d.
Aspergillus oryzae	0	0	0	0	0
Chaetomium globosum	0	0	0	0	0
Coprinopsis cinerea	0	0	0	0	0
Aspergillus nidulans	1	0	0	0	1
Fusarium graminearum	0	0	0	0	0
Laccaria bicolor	0	0	0	0	0
Magnaporthe grisea	0	0	0	0	0
Neurospora crassa	0	0	0	0	0
Stagonospora nodorum	0	0	0	0	0
Phycomyces blakesleeanus	0	0	0	0	0
Podospora anserina	0	0	0	0	0
Saccharomyces cerevisiae	27	1	0	3	23
Schizosaccharomyces pombe	16	1	1	5	9
Sclerotinia sclerotiorum	0	0	0	0	0
Trichoderma reesei	0	0	0	0	0
Ustilago maydis	0	0	0	0	0
Yarrowia lipolytica	2	0	0	0	2
