# Supplementary material for: FUNGIpath: a tool to assess fungal metabolic pathways predicted by orthology
Source: BMC Genomics. 2010 Feb 1;11:81. doi: 10.1186/1471-2164-11-81 (PMC2829015; doi:10.1186/1471-2164-11-81)
Supplement: Additional file 11 — Comparison of data for FUNGIpath genomes. The table provides, for each genome, the number of sequences with complete enzymatic annotation for FUNGIpath, KEGG and Swiss-Prot. [file 1471-2164-11-81-S11.PDF]

Species	Estimated number of CDS	FUNGIpath		KEGG		Swiss-Prot	
		Number of ID with EC	Percent of ID with EC	Number of ID with EC	Percent of ID with EC	Number of ID with EC	Percent of ID with EC
Aspergillus nidulans	9727	1669	17.2%	924	9.5%	149	1.5%
Aspergillus oryzae	12074	1930	16.0%	1094	9.1%	82	0.7%
Batrachochytrium dendrobatidis	8818	1000	11.3%				
Chaetomium globosum	11124	1397	12.6%			22	0.2%
Coprinus cinereus	13544	1405	10.4%			5	0.0%
Fusarium graminearum	11640	1614	13.9%	693	6.0%	41	0.4%
Laccaria bicolor	20000	1392	7.0%	650	3.3%	3	0.0%
Magnaporthe grisea	13146	1540	11.7%	1013	7.7%	30	0.2%
Mycosphaerella graminicola	11395	1513	13.3%			4	0.0%
Neurospora crassa	10082	1277	12.7%	808	8.0%	213	2.1%
Phycomyces blakesleeanus	14792	1619	11.0%			8	0.1%
Podospora anserina	10545	1431	13.6%	633	6.0%	22	0.2%
Puccinia graminis	20567	1149	5.6%				
Saccharomyces cerevisiae	5860	1142	19.5%	1027	17.5%	1163	19.8%
Schizosaccharomyces pombe	5004	981	19.6%	922	18.4%	876	17.5%
Sclerotinia sclerotiorum	14522	1427	9.8%	615	4.2%	4	0.0%
Stagonospora nodorum	16597	1630	9.8%			23	0.1%
Trichoderma reesei	9129	1395	15.3%			21	0.2%
Ustilago maydis	6631	1095	16.5%	725	10.9%	73	1.1%
Yarrowia lipolytica	6520	1207	18.5%	860	13.2%	131	2.0%
Average	11586	1391	13.3%	830	9.5%	159	2.6%
