# Supplementary material for: FUNGIpath: a tool to assess fungal metabolic pathways predicted by orthology
Source: BMC Genomics. 2010 Feb 1;11:81. doi: 10.1186/1471-2164-11-81 (PMC2829015; doi:10.1186/1471-2164-11-81)
Supplement: Additional file 12 — Sources of the genomic data used in FUNGIpath. The respective sequencing centers of each fungal genome are indicated by the url we used to download the primary genomic data. [file 1471-2164-11-81-S12.PDF]

Genome Name	Source	Url
Aspergillus nidulans	Broad Institute	http://www.broad.mit.edu/annotation/genome/aspergillus_nidulans...
Aspergillus oryzae	NITE	ftp://ftp.bio.nite.go.jp/pub/ao/
Batrachochytrium dendrobatidis	Broad Institute	http://www.broad.mit.edu/annotation/genome/batrachochytrium_d...
Chaetomium globosum	Broad Institute	http://www.broad.mit.edu/annotation/genome/chaetomium_globos...
Coprinus cinereus	Broad Institute	http://www.broad.mit.edu/annotation/genome/coprinus_cinereus/I...
Fusarium graminearum	Broad Institute	http://www.broad.mit.edu/annotation/genome/fusarium_graminear...
Laccaria bicolor	JGI	ftp://ftp.jgi-psf.org/pub/JGI_data/Laccaria_bicolor/
Magnaporthe grisea	Broad Institute	http://www.broad.mit.edu/annotation/genome/magnaporthe_grisea...
Mycosphaerella graminicolla	JGI	ftp://ftp.jgi-psf.org/pub/JGI_data/Mycosphaerella_graminicola/v1....
Neurospora crassa	Broad Institute	http://www.broad.mit.edu/annotation/genome/neurospora/Downlo...
Phycomyces blakesleeanus	JGI	ftp://ftp.jgi-psf.org/pub/JGI_data/Phycomyces_blakesleeanus/ann...
Podospora anserina	IGM	http://podospora.igmors.u-psud.fr/download_fr.html
Puccinia graminis	Broad Institute	http://www.broad.mit.edu/annotation/genome/puccinia_graminis/I...
Saccharomyces cerevisiae	Stanford	ftp://genome-ftp.stanford.edu/pub/yeast/data_download/sequence/...
Schizosaccharomyces pombe	SANGER	ftp://ftp.sanger.ac.uk/pub/yeast/pombe/
Sclerotinia sclerotiorum	Broad Institute	http://www.broad.mit.edu/annotation/genome/sclerotinia_sclerotio...
Stagonospora nodorum	Broad Institute	http://www.broad.mit.edu/annotation/genome/stagonospora_nodo...
Trichoderma reesei	JGI	http://genome.jgi-psf.org/Trire2/Trire2.download.html
Ustilago maydis	Broad Institute	http://www.broad.mit.edu/annotation/genome/ustilago_maydis/Do...
Yarrowia lipolytica	Genolevures	http://cbi.labri.u-bordeaux.fr/Genolevures/download.php#yali
